# Predictors of Post-Intensive Care Syndrome in Family Members of Patients With Severe Sepsis

**DOI:** 10.1186/2197-425X-3-S1-A37

**Published:** 2015-10-01

**Authors:** B Matt, D Schwarzkopf, M Fritzenwanger, OW Witte, K Reinhart, CS Hartog

**Affiliations:** Jena University Hospital, Center for Sepsis Control and Care, Jena, Germany; Jena University Hospital, Department of Internal Medicine I, Jena, Germany; Jena University Hospital, Department of Neurology, Jena, Germany; Jena University Hospital, Department of Anesthesiology and Intensive Care Medicine, Jena, Germany

## Introduction

Relatives of patients with severe sepsis have high risk of adverse psychological outcomes. Better knowledge about risk factors is needed.

## Objectives

To predict psychological outcomes in relatives of patients at 90 days after death or discharge.

## Methods

Prospective study on 4 ICUs in one German University hospital (04/2014 - 01/2015). The main relative of consecutive patients with severe sepsis were interviewed by phone at 90 days after patient discharge or death. Posttraumatic stress symptoms (PTSS) were assessed by the Impact of Event Scale (IES), symptoms of anxiety and depression by the Hospital Anxiety and Depression Scale (HADS). Predictors were chosen based on literature, including demographic data, satisfaction with ICU care and information and experience of end-of-life care in the ICU. All patients' health status before severe sepsis and survivors' health status at 90 days were assessed by the relative using the EQ-5D questionnaire. a new item was introduced: feeling overstrained by the ICU experience with rating on a scale from 1 to 10. Linear regression analyses were used to identify predictors in the full sample and among relatives of deceased and surviving patients.

## Results

143 relatives (64% response rate) participated. Fifty (35%) patients died in the ICU, 78 (55%) were alive at the time of the interview. Among relatives, median [IQR] age was 54 [47,63], 73% were female, 43% were spouses and 39% were children of the patient, 78% were legal proxies. After 90 days, 66 relatives (47%) experienced symptoms of PTSS; 55 (39%) and 41 (29%) suffered from symptoms of anxiety and depression, respectively. IES, HADS anxiety or depression scores did not differ between relatives of deceased and surviving patients. By multivariate analyses no item on satisfaction with the ICU experience or the experience of end-of-life care reached significance. Female gender and lower education of relatives were risk factors for some psychological symptoms in the full sample and among relatives of deceased patients. Tracheostomy was a predictor of IES in the full sample (p=.004), treatment on a surgical ICU was a predictor of IES among relatives of deceased patients (p=.004). The degree of feeling overstrained by the ICU experience was a predictor of IES, HADS anxiety and depression in the full sample as well as in the subsamples of relatives of deceased and surviving patients (p≤.021). The difference in EQ-5D health status predicted IES and HADS depression among surviving patients (p≤.049).

## Conclusions

Feeling overstrained during the ICU stay might be the strongest predictor of relatives' psychological symptoms after three months. Tracheostomy in the ICU might be a predictor for PTSS. Both should be investigated in prospective longitudinal or interventional studies to better assess and prevent relatives' psychological burden after their ICU experience.

## Grant Acknowledgment

German Ministry of Education and Research, grant no. 01 E0 1002

